# Linking Ecosystem-Service Perceptions and Wool Valorisation in Alpine Sheep Farming Systems

**DOI:** 10.3390/ani16172649

**Published:** 2026-08-24

**Authors:** Chiara Costamagna, Valentina Maria Merlino, Alessandro Petrontino, Alessandra Degli Esposti, Paolo Cornale, Danielle Borra, Luca Maria Battaglini

**Affiliations:** 1 Department of Agricultural, Forest and Food Sciences, University of Turin, Largo Paolo Braccini 2, 10095 Grugliasco, Italy; c.costamagna@unito.it (C.C.); alessandra.degliesposti69@gmail.com (A.D.E.); paolo.cornale@unito.it (P.C.); danielle.borra@unito.it (D.B.); luca.battaglini@unito.it (L.M.B.); 2 Department of Soil, Plant and Food Sciences, University of Bari Aldo Moro, Via Amendola 165/A, 70126 Bari, Italy; alessandro.petrontino@uniba.it

**Keywords:** alpine sheep farming, cultural ecosystem services, local wool product, wool comfort, sustainability, sustainable rural development

## Abstract

Wool is often regarded as a low-value by-product of Alpine sheep farming, despite its potential to convey the ecosystem services (ESs) generated by these systems. This study examines how awareness and perception of these services are associated with consumer attitudes towards locally produced wool in the Lanzo Valleys in Northwestern Italy. A convenience sample of 400 adults familiar with the study area completed an online questionnaire. Principal Component Analysis identified two orientations towards wool products—Sustainability Orientation and Product-Value Orientation—which together explained 69.33% of the variance. A TwoStep Cluster Analysis based on the component scores identified four profiles within the study sample: Sustainability-sensitive (22%), Disinterested (14.25%), Eco-aesthetic (36%), and Aesthetically aware (27.75%). Sustainability-oriented respondents attributed greater importance to ES related to biodiversity, native-breed conservation and animal welfare, whereas aesthetic and experiential attributes represented an additional, distinct dimension of product evaluation. Residence status was not significantly associated with cluster membership. Given the non-probabilistic sampling design, these profiles should be interpreted as exploratory attitudinal patterns. The findings may inform differentiated communication and wool-valorisation strategies linking environmental, cultural, aesthetic and functional values.

## Simple Summary

Mountain sheep farming provides important socio-cultural and environmental benefits, while wool is increasingly regarded as a low-value material. This study investigated whether perceptions of the environmental and socio-cultural benefits associated with Alpine sheep farming are related to attitudes towards locally produced wool, using the Lanzo Valleys in Piedmont as a case study. Because participants were recruited through an online convenience sample, the findings reflect associations rather than causal effects and should not be generalised to the wider population. An online questionnaire was completed by 400 adults familiar with the study area. Principal Component Analysis and TwoStep Cluster Analysis identified four attitudinal profiles that differed in their emphasis on sustainability and product-value attributes. Residence in or outside the Lanzo Valleys was not significantly associated with cluster membership. The findings suggest that communication linking wool to ecosystem services, together with product quality, aesthetics and comfort, may support future strategies for the valorisation of local wool and the diversification of mountain farms.

## 1. Introduction

Mountain livestock farming systems are increasingly expected to reconcile agricultural production with biodiversity conservation, landscape management, and the provision of ecosystem services. However, socio-economic pressures, rural depopulation, global market competition, and climate change are undermining the long-term viability of these systems across many European mountain regions [1,2]. As their productive function alone is often insufficient to ensure economic sustainability, their future increasingly depends on diversification strategies capable of valorising the multiple environmental and cultural benefits they generate.

The reduction in farming activities has generated consequences that extend beyond agricultural production, affecting landscape management, ecosystem services, biodiversity conservation, cultural heritage, place identity reflection, and, in addition, the attractiveness of rural tourism destinations [3,4,5,6,7].

Within Alpine farming systems, sheep husbandry represents a multifunctional activity capable of generating economic, environmental, and socio-cultural benefits [8]. In addition to producing food and fibre, sheep farming contributes to maintaining traditional landscapes, preserving local ecological knowledge, supporting biodiversity, and sustaining cultural identities that are increasingly recognised as strategic resources for rural development.

Among the most affected products is Italian wool. Once considered an important economic resource, wool is now frequently treated as a low-value by-product or even as a disposal cost for farmers due to growing competition from imported fibres and the contraction of domestic processing chains [9,10,11]. This situation is particularly critical in mountain areas, where small-scale sheep farms often struggle to recover shearing costs despite the environmental and cultural value associated with wool production. According to the Millennium Ecosystem Assessment [12] and TEEB [13], ecosystem services can be organised into broad categories including provisioning, regulating, habitat/biodiversity and cultural services. Empirical studies have shown that Alpine and mountain sheep farming can contribute to all of these categories [14,15,16,17]. Cultural services are particularly relevant in mountain regions because they contribute to place identity, the preservation of local traditions, aesthetic appreciation of landscapes, environmental education and tourism experiences.

In the Lanzo Valleys, sheep farming has historically played a significant role in shaping local landscapes and livelihoods. Seasonal grazing practices, traditional pastoral knowledge, and wool-related handicrafts have contributed to the cultural identity of the area. Furthermore, the presence of local and regional sheep breeds represents an important component of livestock genetic resources, strengthening the connection between farming systems, biodiversity conservation, and territorial heritage. The Lanzo Valleys, in the Northwestern Italian Alps, represent a particularly suitable context for investigating these relationships. The area combines rich natural resources and long-standing pastoral traditions with increasing challenges, including rural abandonment, forest encroachment, and the need for new forms of sustainable economic development [18]. The area is currently facing challenges common to many Alpine territories, including demographic decline, land abandonment, reduced agricultural profitability, and the need to diversify farm income sources. In this context, the valorisation of locally produced wool may represent a potential strategy to enhance the multifunctionality and resilience of mountain farming systems while simultaneously enriching tourism experiences [19,20,21]. Evidence from other Alpine regions suggests that strategies focusing on local breeds and territorial identity can support the resilience of small ruminant farms, although a lack of infrastructure for wool valorisation itself has been identified as a limiting factor [22]. Direct empirical evidence on whether wool valorisation specifically produces these outcomes in the Lanzo Valleys remains limited, representing one of the gaps this study aims to address. In addition, although ecosystem services generated by mountain livestock systems have been widely investigated [16,23,24], considerably less attention has been devoted to understanding whether public recognition of these services can increase the perceived value of agricultural by-products. Likewise, studies on wool have mainly focused on textile properties, supply chains or consumer preferences, whereas the relationship between ecosystem-service awareness and the valorisation of locally produced wool remains largely unexplored.

At the same time, the economic marginalisation of wool production has encouraged researchers and practitioners to explore innovative pathways for wool valorisation capable of generating additional value for mountain farms and rural communities. In this perspective, understanding how consumers perceive both wool products and the ecosystem services associated with sheep farming may provide useful insights for strengthening the multifunctionality and resilience of Alpine farming systems.

Furthermore, cultural ecosystem services have increasingly been recognised as important drivers of destination attractiveness, place attachment and rural development. However, little is known about how awareness and perception of these services are associated with attitudes towards wool produced within Alpine livestock systems and with the broader contribution of sheep farming to territorial sustainability.

Transforming low-value agricultural by-products into territorial assets requires more than technological innovation. It also depends on consumers recognising the environmental, cultural and social benefits embedded in those products. To address these knowledge gaps, this study investigates the relationship between perceptions of ecosystem services, attitudes towards local wool, and consumer segmentation in the Lanzo Valleys, a mountain area characterised by a strong pastoral tradition and growing interest in sustainable tourism development.

Specifically, this study addresses the following research questions:

RQ1. How do consumers perceive the ecosystem services generated by Alpine sheep farming systems?

RQ2. To what extent are attitudes towards wool derived from Alpine sheep farming systems associated with differing levels of awareness and perception of ecosystem services?

RQ3. What implications do different consumer profiles have for the valorisation of local wool and the sustainability of Alpine sheep farming systems?

Accordingly, this study aims to investigate how consumers perceive the ecosystem services generated by Alpine sheep farming systems and how such perceptions are associated with attitudes towards locally produced wool. Furthermore, the study identifies distinct attitudinal profiles within the sample and discusses how these findings may inform strategies for farm diversification and the valorisation of mountain livestock systems.

## 2. Materials and Methods

### 2.1. Study Area

The study was conducted in the Lanzo Valleys, a mountain area located in the Northwestern Italian Alps (Piedmont Region, Italy), comprising the Viù, Ala and Grande valleys. The area extends over approximately 695 km^2^ and includes 19 municipalities. According to the Regione Piemonte agricultural information system (SIAP–Anagrafe Agricola Unica), data updated on 11 November 2022 indicate 303 agricultural holdings in the Lanzo Valleys, of which 234 included livestock activities. Sheep farming accounted for 38 holdings and a total of 3182 sheep [25]. These data confirm the continuing relevance of sheep farming within the local agricultural sector and support the selection of the Lanzo Valleys as a case study for investigating perceptions of Alpine sheep farming and locally produced wool.

Given the established presence of sheep farming in the area and the current marginal economic value of wool, the Lanzo Valleys provide a relevant case study for investigating how consumers perceive the ecosystem services associated with Alpine sheep farming and how these perceptions are related to attitudes towards locally produced wool.

### 2.2. Survey Design and Data Collection

Data were collected through a structured online questionnaire administered between March and September 2025 using the Google Forms platform (Google LLC, Mountain View, CA, USA). The questionnaire targeted consumers over 18 yrs. familiar with the study area and included both residents and non-residents of the Lanzo Valleys. Because the objective of the study was to investigate consumer perceptions of wool and ecosystem services rather than compare residents and visitors, residence was treated as a socio-demographic characteristic describing the sample rather than as the primary analytical variable. Participation was voluntary and anonymous. Respondents who did not meet these eligibility criteria were excluded from the analysis. The questionnaire was disseminated through local Facebook groups and social-media communities related to the Lanzo Valleys. Consequently, the study adopted a non-probabilistic convenience sampling strategy. Recruitment through social-media groups may have favoured respondents already connected to the area or interested in local products, sustainability, rural development, or pastoral activities. For this reason, the results should be interpreted as evidence of attitudinal patterns within a self-selected sample and should not be generalised to the wider population of residents, visitors, or Alpine consumers.

Participation was voluntary and anonymous. Before completing the questionnaire, respondents were informed about the objectives of the study and consented to the anonymous use of their responses for research purposes. Ethical approval was granted by the Bioethics Committee of the University of Turin (Protocol No. 0513153, 3 October 2022), in accordance with the principles of the Declaration of Helsinki.

The questionnaire was developed based on previous studies investigating ecosystem-service valuation, sustainable consumption and consumer behaviour related to agricultural and livestock products [16,26,27]. The instrument consisted of four sections: (1) socio-demographic characteristics (gender, age, educational level, annual family income, residence in the Lanzo Valleys and size of the municipality of residence); (2) consumer concerns regarding environmental issues; (3) wool purchasing habits and product preferences; and (4) knowledge and perception of ecosystem services generated by mountain sheep farming systems. The complete wording of the questionnaire items and the corresponding response options are reported in Tables S1 and S2 of the Supplementary Materials.

The variable ‘knowledge of the wool production process’ was coded as an ordinal three-category indicator (0 = ‘No’, 1 = ‘Yes, approximately’, and 2 = ‘Yes’). Its inclusion in the PCA therefore represents an approximation and is acknowledged as a methodological limitation. Parametric procedures have nevertheless been shown to be relatively robust to moderate departures from strict interval-level assumptions in large samples [28]. The contribution of this variable was therefore interpreted cautiously, particularly because it showed the lowest loading among the variables included in the analysis (0.509).

### 2.3. Data Analysis

Descriptive statistics were initially calculated to summarise the socio-demographic characteristics of the sample and the distribution of questionnaire responses.

A Principal Component Analysis (PCA) with Varimax rotation was conducted to identify the main latent dimensions underlying respondents’ orientations towards wool products. The analysis included 11 purchasing-attribute items and the coded indicator of knowledge of the wool production process. Because the questionnaire was multidimensional rather than a single unidimensional psychometric scale, a global Cronbach’s alpha across all questionnaire items was not considered appropriate. Internal consistency was instead assessed for the item sets defining the extracted components, while the suitability of the correlation matrix for component extraction was evaluated using the Kaiser–Meyer–Olkin (KMO) measure and Bartlett’s test of sphericity.

The suitability of the data for component extraction was assessed using the Kaiser–Meyer–Olkin (KMO) measure of sampling adequacy and Bartlett’s test of sphericity. Components with eigenvalues greater than 1.0 were retained according to the Kaiser criterion [29]. Factor loadings greater than 0.30 were considered meaningful for interpretation. Cronbach’s alpha was 0.952 for the Sustainability Orientation component and 0.817 for the Product-Value Orientation component, indicating satisfactory to excellent internal consistency.

Component scores obtained from the PCA were subsequently used as continuous summary variables in a TwoStep Cluster Analysis to identify respondent profiles according to their orientations towards local wool products. Although TwoStep Cluster Analysis is frequently used because it can accommodate mixed data types, the procedure can also be applied to exclusively continuous variables using Euclidean distance [30], which was the option used in this study. The method was retained for this exploratory segmentation because it allows the number of clusters to be selected automatically using information-theoretic criteria. Candidate solutions ranging from one to 15 clusters were evaluated. The greatest improvement occurred when moving from three to four clusters: AIC decreased from 543.793 to 350.103 (ΔAIC = −193.689), while BIC decreased from 591.690 to 413.967 (ΔBIC = −177.723). The four-cluster solution also showed the largest ratio of distance measures (2.609), supporting a clear separation relative to the preceding solution.

Differences among clusters were assessed using one-way Analysis of Variance (ANOVA) for variables measured on seven-point Likert scales. Although Likert-scale responses are ordinal in nature, ANOVA was considered appropriate because of the relatively large sample size (*N* = 400) and the demonstrated robustness of parametric procedures under moderate violations of normality assumptions. Homogeneity of variances was evaluated before conducting post hoc comparisons [31].

When significant differences were detected, Tukey’s Honestly Significant Difference (HSD) test was applied for pairwise comparisons. Associations involving categorical variables were evaluated using Chi-square tests [32], with Cramér’s V reported as an effect-size measure for the socio-demographic comparisons. Statistical significance was established at *p* < 0.05. Because cluster membership was generated from the PCA scores, comparisons using the component scores themselves were treated as descriptive characteristics of the solution, whereas variables not used in the PCA or clustering procedure were used for external characterisation of the derived profiles.

All analyses were performed using IBM SPSS Statistics version 28 (IBM Corp., Armonk, NY, USA).

## 3. Results

### 3.1. Socio-Demographic Characteristics of Interviewees

A total of 400 valid questionnaires were included in the analysis. The sample was predominantly female (78.8%), and more than half of the participants were aged between 46 and 65 years. Approximately 42% of respondents lived in the Lanzo Valleys, whereas 58% lived outside the study area. Most respondents had completed upper-secondary education or held a university degree. The complete distribution by gender, age, educational level, annual family income, residence and municipality size is reported in Supplementary Table S3. These characteristics should be considered when interpreting the findings because the sample was recruited through a non-probabilistic online procedure and was not designed to reproduce the socio-demographic structure of the wider Alpine population.

### 3.2. Purchasing Choice Orientations

Principal Component Analysis identified two distinct dimensions underlying respondents’ orientations towards wool products and environmental sustainability. Together, these components explained 69.33% of the total variance. The data showed excellent sampling adequacy and a suitable correlation structure for component extraction (KMO = 0.911; Bartlett’s test: χ^2^ = 4492.064, *p* < 0.001) (Table 1). Internal consistency was excellent for Sustainability Orientation (Cronbach’s α = 0.952) and satisfactory for Product-Value Orientation (Cronbach’s α = 0.817).

The first principal component (PC1, explaining 59.14% of the variance) was termed Sustainability Orientation. It was characterised by high loadings for environmental sustainability, animal welfare, respect for working conditions and fair pay, health safety, product quality and Made in Italy. Thus, the component combined environmental and ethical attributes with quality-related product evaluations. This empirical pattern is compatible with halo effects described in the corporate-social-responsibility and ethical-consumption literature, whereby socially responsible production cues can influence broader product evaluations [33]. The second component (PC2, explaining a further 10.19% of the variance) was termed Product-Value Orientation and was characterised by price, aesthetics, comfort, originality, brand and knowledge of the wool production process. It therefore captured predominantly intrinsic, experiential and market-related product attributes rather than sustainability considerations.

### 3.3. Cluster Characterisation

The TwoStep Cluster Analysis identified four clusters within the study sample, as shown in Table 2. These clusters were derived exclusively from the two PCA scores and should therefore be interpreted as exploratory attitudinal profiles within the present non-probability sample rather than as generalisable consumer typologies. The Sustainability-sensitive profile (22%) combined a positive Sustainability Orientation with comparatively low Product-Value Orientation. The Disinterested profile (14.25%) scored low on both dimensions. The Eco-aesthetic profile (36%) combined positive scores on both sustainability and product-value dimensions, whereas the Aesthetically aware profile (27.75%) showed the highest Product-Value Orientation and a modestly positive Sustainability Orientation.

All variables used in the subsequent characterisation—socio-demographic characteristics, environmental concerns, purchasing habits, detailed comfort indicators, and awareness and perception of ecosystem services—were external to the PCA and cluster-formation process. They were therefore used to characterise and externally corroborate the derived typology rather than being definitionally embedded in it.

Socio-demographic characteristics were compared across the four clusters using Chi-square tests (Table 3). Age group was the only socio-demographic variable significantly associated with cluster membership (χ^2^ = 39.560, df = 15, *p* = 0.0005; Cramér’s V = 0.182), corresponding to a small-to-moderate association. The Aesthetically aware profile contained a comparatively larger share of younger respondents aged 18–35, whereas respondents aged 36–55 were proportionally more represented in the Disinterested profile. The Sustainability-sensitive profile included a comparatively larger share of respondents aged 56–65, while the Eco-aesthetic profile showed the highest incidence of respondents aged 65 years and over. No statistically significant associations were found for gender, educational level, annual family income, size of the municipality of residence, or residence in versus outside the Lanzo Valleys.

Residence status was not significantly associated with cluster membership (χ^2^ = 3.123, df = 3, *p* = 0.373), and the corresponding effect size was very small (Cramér’s V = 0.088). This result indicates that residence in or outside the Lanzo Valleys was not a major determinant of the attitudinal profiles identified in this sample.

### 3.4. Concerns Towards Environmental Issues

Significant differences emerged among the four clusters regarding the environmental concerns perceived by individuals (see Table 4). While the majority of respondents expressed high levels of concern regarding pollution, waste and resource depletion, the Disinterested cluster consistently exhibited the lowest mean scores across all environmental topics (*p* < 0.001). The highest levels of concern were observed in the Eco-aesthetic and Aesthetically aware clusters, particularly with regard to air, water and soil pollution, increasing waste and climate change.

### 3.5. Purchasing Habits, Choices and Preferences of Wool Products

Purchasing frequency and product choice varied significantly across clusters (Table 5). The Disinterested cluster, despite its low engagement with sustainability, reported the highest frequency of wool purchases (“Often” = 19.3%). In contrast, Sustainability-sensitive consumers showed the lowest frequency (“Never” = 10.2%).

The most purchased wool products were clothes and personal accessories. Although the Chi-square analysis revealed no statistically significant differences between clusters in the type of wool products purchased, notable patterns emerged. Eco-aesthetic consumers most frequently purchased woollen garments, blankets, pillows and accessories, while Sustainability-sensitive consumers reported the highest purchase of woollen mattresses. The largest proportion of woollen carpets was purchased by Disinterested respondents.

Regarding comfort indicators (Table 5), all clusters except Disinterested rated softness, warmth and non-irritating feel as highly important. The Aesthetically aware cluster reported the highest mean scores for washability at high temperatures and non-stinging feel, reflecting a pragmatic and sensory-driven perception of comfort.

### 3.6. Perception of Ecosystem Services Provided by Mountain Sheep Farming

Awareness of the term “Ecosystem Services” was unevenly distributed (Table 6). While most respondents had heard of the term, only a minority could accurately define it. The Disinterested cluster paradoxically showed the highest percentage of individuals claiming familiarity, though their subsequent ratings indicated weak conceptual understanding.

In response to the first research question (RQ1), the total sample (*N* = 400) was considered. This group consistently attributed high importance to the ecosystem services associated with Alpine sheep farming (see Table S4 in the Supplementary Materials), regardless of cluster affiliation. The average scores (on a scale of 0 to 6) were particularly high: 4.74 for provisioning services, 4.75 for regulating services, 4.82 for habitat and biodiversity services, 4.79 for cultural services, and 4.82 for animal welfare.

The ANOVA results (see Table S4 in the Supplementary Materials) revealed significant differences (*p* < 0.001) between clusters for all ES categories. Sustainability-sensitive consumers consistently reported the highest scores across all provisioning ecosystem services, whereas the disinterested cluster attributed substantially lower importance. Respondents who were Eco-aesthetic or Aesthetically aware occupied intermediate positions, although their evaluations remained significantly higher than those of the disinterested segment.

The largest differences between consumer groups emerged in relation to biodiversity conservation and the preservation of native breeds, suggesting that these ecosystem services are particularly sensitive to individual sustainability attitudes.

Cultural ecosystem services received consistently high ratings from Sustainability-sensitive, Eco-aesthetic, and Aesthetically aware consumers. This finding suggests that landscape appreciation and cultural heritage may be important ways in which consumers value wool from Alpine sheep farming systems.

Finally, animal welfare emerged as one of the ecosystem services showing the greatest discrimination among consumer segments, reinforcing its role as a central element in consumers’ evaluation of mountain livestock systems.

Overall, the results indicate that consumer segmentation is strongly associated with perception of ecosystem services, with sustainability-oriented consumers consistently assigning greater value to the environmental, cultural, and animal welfare functions performed by Alpine sheep farming systems.

## 4. Discussion

### 4.1. Perception of Ecosystem Services in Alpine Sheep Farming Systems (RQ1)

The present study explored how respondents perceive the ecosystem services generated by Alpine sheep farming systems, with specific attention to the role of local wool as a multifunctional resource. Ecosystem-service recognition differed across the four exploratory attitudinal profiles, indicating that Sustainability Orientation was associated with how respondents evaluated the broader functions performed by mountain livestock systems.

Overall, respondents attributed high importance to most ecosystem services associated with sheep farming, particularly those related to the preservation of native breeds, landscape maintenance, biodiversity conservation and animal welfare. These results are consistent with the multifunctional role attributed to mountain livestock systems in previous studies, whose contributions extend beyond agricultural production to environmental stewardship, cultural heritage preservation and socio-ecological sustainability [8,16,34,35].

The preservation of native sheep breeds, in particular, has been shown to add value to Alpine agroecosystems more broadly [22].

The high scores assigned to cultural ecosystem services are particularly notable. Respondents recognised the contribution of sheep farming to maintaining traditional landscapes, preserving local traditions and supporting the identity of mountainous regions. These findings are consistent with previous studies that have emphasised the significance of cultural ecosystem services in shaping public appreciation of mountain agriculture and rural landscapes [24,36]. For example, another typical Italian livestock system explored by [16] emerged, in which cultural and landscape services were the most highly valued by consumers, demonstrating that the local shepherding model has formed the basis of the local cultural identity. This model provides landscape maintenance and care, contributes to the protection of the territory, animal and vegetable biodiversity, and ensures employment for thousands of people, enhancing cultural identity in the Alps and safeguarding the typical landscape. The findings suggest that local populations and visitors increasingly perceive pastoral systems as providing broader societal benefits as well as being productive activities.

Residence status was not significantly associated with cluster membership (χ^2^ = 3.123, *p* = 0.373; Cramér’s V = 0.088). This suggests that, within the present sample, attitudes towards local wool and ecosystem services were not primarily structured by residence in or outside the Lanzo Valleys. The identified profiles should therefore be interpreted principally as attitudinal patterns rather than as residence-based groups [37]. Traditionally, ecosystem services have often been discussed as public environmental benefits requiring policy support. The present findings suggest that consumers may also connect these services with the perceived value of agricultural products. In Alpine sheep farming, locally produced wool may therefore provide a tangible means of communicating biodiversity conservation, landscape maintenance, cultural heritage and animal-welfare values, although the present cross-sectional study cannot demonstrate that such communication causes increased purchasing or farm diversification.

### 4.2. Relationship Between Ecosystem-Service Awareness and Attitudes Towards Local Wool (RQ2)

The results showed an association between ecosystem-service awareness and respondents’ attitudes towards local wool products. In particular, Sustainability-sensitive respondents attributed greater importance to provisioning, regulating, habitat-related, cultural and animal-welfare ecosystem services. These findings indicate that awareness of the broader contributions of sheep farming is associated with a greater appreciation of local wool as a product linked to place and sustainability. This is consistent with evidence that environmental knowledge and concern are related to pro-environmental and sustainable consumption orientations more broadly [38]. However, ecosystem-service awareness alone does not explain respondents’ evaluations of wool products. The Eco-aesthetic and Aesthetically aware profiles show that aesthetic, sensory and experiential attributes also represent important dimensions of stated preference, consistent with research on sustainable fashion in which environmental concerns coexist with expectations regarding comfort, design, quality and personal identity [39,40,41,42].

The survey also revealed that stated Sustainability Orientations did not correspond perfectly to self-reported purchase frequency. For example, respondents in the Disinterested profile reported purchasing wool despite assigning comparatively low importance to sustainability-related characteristics. Because the study measured self-reported frequency rather than observed purchasing behaviour or willingness to pay, this pattern should not be interpreted as evidence of an attitude–behaviour gap [43,44,45]. It instead indicates that wool purchases may reflect motivations not captured by Sustainability Orientation alone, such as habit, functional needs or product availability.

Although wool is often regarded as a low-value by-product within contemporary livestock production systems, the present findings suggest that consumers may attribute greater value to wool when they recognise the ecosystem services embedded in its production. This interpretation contributes to the growing literature on multifunctional agriculture by suggesting that ecosystem services may increase the perceived value of agricultural by-products rather than remaining abstract environmental concepts.

These findings build on previous research into ecosystem services by demonstrating that public appreciation of the provisioning, regulating and cultural functions of ecosystems is closely linked to broader consumer value orientations, rather than to product evaluation alone. Furthermore, the results of our research suggest that wool valorisation strategies should consider a range of factors, rather than relying exclusively on sustainability narratives, to achieve effective outcomes. Instead, communication efforts should integrate environmental, cultural, aesthetic, and functional dimensions in order to appeal to the diverse motivations of different consumer groups.

### 4.3. Consumer Profiles and Implications for Wool Valorisation (RQ3)

Due to the non-probabilistic and self-selected nature of the sample (described in Section 2.2), the four profiles identified here should be considered as attitudinal patterns observed within this particular sample instead of as validated or generalisable consumer typologies. Furthermore, although the four clusters are treated here as distinct consumer segments, they should not be interpreted as fully independent behavioural groups; rather, they represent a typology derived from PCA-based orientations and corroborated by external, non-PCA variables, within the limits imposed by the non-probabilistic sampling strategy. With this consideration in mind, the cluster analysis identified four distinct consumer profiles characterised by different combinations of sustainability and product value orientation. Taken together, these profiles offer preliminary, exploratory insights that could inform, but not directly determine, how local wool can be marketed and positioned in mountain regions.

The Sustainability-sensitive profile showed a strong appreciation of ecosystem services and ethical production attributes. This suggests that communication emphasising sustainability, biodiversity conservation, animal welfare and transparent production may be particularly relevant to respondents with this orientation. Such strategies should be regarded as hypotheses for future market testing rather than as demonstrated drivers of purchasing behaviour. Social-media platforms have already been shown to play a role in constructing sustainability-oriented wool narratives [46].

In contrast, the Aesthetically aware cluster was more sensitive to design, originality, and sensory qualities, highlighting the importance of developing contemporary products and experiential marketing approaches.

The Eco-aesthetic cluster, representing the largest segment of the sample, is particularly promising for initiatives promoting wool. Interestingly, the Eco-aesthetic segment indicates that sustainability concerns and aesthetic appreciation are not mutually exclusive but rather complementary dimensions influencing product evaluation. Conversely, the Disinterested segment suggests that sustainability messages alone are unlikely to increase appreciation of locally produced wool among all consumers, highlighting the need for differentiated communication strategies.

Overall, the identified profiles highlight the need for differentiated communication and marketing strategies that can address multiple dimensions of value. Such an approach could increase consumer engagement with local wool whilst raising awareness of the ecosystem services generated by mountain sheep farming systems [47].

### 4.4. Implications for Alpine Sheep Farming Systems

The findings have broader implications for mountain farming systems beyond the wool sector. Although wool currently generates limited economic returns in many mountain regions, the survey respondents associated wool production with a variety of ecosystem services and territorial values. This suggests that wool could be a valuable, underutilised resource that could contribute to the multifunctionality of mountain farming systems.

Rather than being regarded solely as a fibre commodity, local wool can be seen as an asset embedded within broader socio-ecological systems. This is consistent with broader trends in the valorisation of natural fibres within sustainable textile innovation [48,49] and with design strategies aimed at repositioning traditional materials within contemporary sustainable production and consumption systems [50]. Strengthening the links between sheep farmers, local processors, craftspeople, retailers and tourism operators could transform wool from a low-value by-product into a means of communicating environmental stewardship, cultural heritage and local identity.

Furthermore, the strong appreciation expressed for native breeds, landscape conservation and traditional pastoral practices points to opportunities to link wool valorisation strategies with biodiversity conservation and rural development policies. Regional branding, certification schemes, educational activities and experiential tourism programmes are examples of initiatives that could increase the perceived value of wool whilst supporting farm diversification and resilience. The potential contribution of wool valorisation to the sustainability of mountain livestock systems has also been highlighted in previous research focusing on local sheep breeds in Italy. For example, studies on the Gentile di Puglia sheep breed (typical in Southern Italy regions) demonstrated that the development of innovative wool supply chains could improve farm profitability while supporting the conservation of local genetic resources and the management of marginal rural territories. In that context, wool was proposed not only as a textile fibre but also as a strategic resource capable of generating economic, environmental, and socio-cultural benefits through the creation of regional value chains and territorial branding initiatives [51].

Our findings are consistent with this perspective. Respondents belonging to the Sustainability-sensitive and Eco-aesthetic clusters attributed high importance to ecosystem services related to biodiversity conservation, preservation of native breeds, landscape maintenance, and cultural heritage. These results suggest that consumers may recognise values that extend beyond the physical characteristics of wool products and encompass the broader socio-ecological functions performed by sheep farming systems. Consequently, local wool could contribute to strengthening the economic viability of mountain farms while simultaneously supporting ecosystem-service provision and the conservation of traditional pastoral systems.

Increasing consumers’ awareness of ecosystem services may contribute to improving the economic valorisation of agricultural by-products while simultaneously reinforcing the multifunctional role of livestock farming. From a farming-systems perspective, these results reinforce the importance of considering economic, environmental, cultural, and social dimensions simultaneously when developing strategies aimed at supporting the long-term viability of mountain livestock systems.

From a policy perspective, the results suggest that initiatives supporting mountain livestock farming should combine technical and economic measures with communication strategies to raise public awareness of ecosystem services. These approaches could boost the market value of wool while also promoting biodiversity conservation, maintaining the landscape and preserving cultural heritage.

### 4.5. Limitations and Future Research

Several limitations should be acknowledged. First, the study was based on a non-probability convenience sample recruited via social media, which limits the generalisability of the findings. The sample was strongly female-dominated and was not balanced across age, educational, income and settlement-size categories; these socio-demographic imbalances may have influenced the observed responses. Consequently, the identified profiles should be interpreted as exploratory attitudinal patterns within this specific sample rather than as representative consumer typologies for the wider Alpine population.

Secondly, the study relied on self-reported perceptions and purchasing habits rather than observed behaviour or measures of willingness to pay. Therefore, the identified relationships should not be interpreted as evidence of actual market behaviour. Future studies could integrate behavioural experiments, willingness-to-pay assessments and longitudinal approaches to better understand how perceptions translate into purchasing decisions. Finally, further research should explore the role of local wool within broader transitions towards sustainable farming systems, circular bioeconomy initiatives, and rural-development and participatory governance strategies in mountain regions.

## 5. Conclusions

This study identified two main dimensions associated with respondents’ attitudes towards local wool—Sustainability Orientation and Product-Value Orientation—and four exploratory attitudinal profiles within the investigated sample. These profiles ranged from low engagement to combined appreciation of sustainability and experiential product values. Perceptions of ecosystem services were associated with these orientations: respondents with stronger Sustainability Orientations generally attributed greater importance to biodiversity conservation, native-breed preservation, cultural services and animal welfare. Given the exploratory design and the non-probabilistic convenience sample, the findings should not be interpreted as causal or representative of the wider Alpine population. Nevertheless, the results contribute to the literature on multifunctional farming systems by showing that ecosystem-service perceptions are associated with distinct orientations towards locally produced wool. Rather than demonstrating that recognition of ecosystem services directly generates economic opportunities, the findings identify attitudinal patterns that may inform future communication, market-testing and wool-valorisation strategies integrating environmental, cultural, aesthetic and functional values.

## Figures and Tables

**Table 1:** Rotated components matrix.

Variables	Sustainability Orientation	Product-Value Orientation
Price		0.622
Quality	0.749	
Aesthetics		0.728
Comfort		0.619
Made in Italy	0.702	
Originality of the garment		0.668
Brand		0.566
Health safety	0.901	
Environmental sustainability	0.914	
Respect for working conditions and fair pay	0.892	
Respect for animal welfare	0.916	
Knowledge of wool production process		0.509
Cronbach’s α	0.952	0.817

**Table 2:** Cluster definition.

Principal Component	Sustainability-Sensitive	Disinterested	Eco- Aesthetic	Aesthetically Aware	F	*p*-Value
Product-Value Orientation	−1.147	−0.776	0.096	1.182	438.246	<0.001
Sustainability Orientation	0.500	−2.042	0.360	0.185	318.305	<0.001
% of total sample	22%	14.25%	36%	27.75%		

**Table 3:** Association between cluster membership and socio-demographic characteristics.

Variable	χ^2^	df	*p*-Value	Cramér’s V
Gender	12.040	6	0.061	0.123
Age group	39.560	15	0.0005	0.182
Educational level	16.587	12	0.166	0.118
Annual family income	16.802	12	0.157	0.118
Size of municipality of residence	23.547	18	0.170	0.140
Residence (Lanzo Valleys/outside)	3.123	3	0.373	0.088

**Table 4:** Multi-factor ANOVA results for degree of environmental concern by cluster.

Environmental Topic	Cluster	Mean	SD	F	*p*-Value
Daily use of chemicals	Sustainability-sensitive	4.74 ^b^	1.823	19.859	<0.001
	Disinterested	3.04 ^a^	1.812		
	Eco-aesthetic	5.03 ^b^	1.386		
	Aesthetically aware	4.55 ^b^	1.828		
Consumer habits	Sustainability-sensitive	4.68 ^b^	1.678	23.416	<0.001
	Disinterested	2.84 ^a^	1.850		
	Eco-aesthetic	4.85 ^b^	1.401		
	Aesthetically aware	4.68 ^b^	1.647		
The environmental impact of transport	Sustainability-sensitive	4.43 ^b^	1.687	19.263	<0.001
	Disinterested	2.88 ^a^	1.862		
	Eco-aesthetic	4.68 ^b^	1.294		
	Aesthetically aware	4.50 ^b^	1.634		
Deforestation	Sustainability-sensitive	4.77 ^b^	1.818	23.894	<0.001
	Disinterested	3.05 ^a^	1.777		
	Eco-aesthetic	4.98 ^b^	1.293		
	Aesthetically aware	4.95 ^b^	1.510		
Food handling and production techniques	Sustainability-sensitive	4.72 ^b^	1.906	19.329	<0.001
	Disinterested	3.02 ^a^	1.913		
	Eco-aesthetic	4.96 ^b^	1.394		
	Aesthetically aware	4.73 ^b^	1.705		
The increase in waste	Sustainability-sensitive	4.88 ^b^	1.707	30.079	<0.001
	Disinterested	3.07 ^a^	1.850		
	Eco-aesthetic	5.20 ^b^	1.186		
	Aesthetically aware	4.98 ^b^	1.414		
The depletion of natural resources	Sustainability-sensitive	4.76 ^b^	1.702	25.696	<0.001
	Disinterested	3.14 ^a^	1.894		
	Eco-aesthetic	5.12 ^b^	1.156		
	Aesthetically aware	4.95 ^b^	1.458		
Soil pollution	Sustainability-sensitive	4.93 ^b^	1.581	25.853	<0.001
	Disinterested	3.05 ^a^	1.977		
	Eco-aesthetic	5.13 ^b^	1.270		
	Aesthetically aware	4.86 ^b^	1.649		
Air pollution	Sustainability-sensitive	5.03 ^b^	1.636	30.010	<0.001
	Disinterested	3.23 ^a^	1.927		
	Eco-aesthetic	5.27 ^b^	1.130		
	Aesthetically aware	5.16 ^b^	1.365		
Water pollution	Sustainability-sensitive	4.97 ^b^	1.572	29.428	<0.001
	Disinterested	3.12 ^a^	1.946		
	Eco-aesthetic	5.22 ^b^	1.219		
	Aesthetically aware	5.05 ^b^	1.458		
Climate change	Sustainability-sensitive	4.36 ^b^	2.069	20.084	<0.001
	Disinterested	3.00 ^a^	2.087		
	Eco-aesthetic	4.93 ^b^	1.408		
	Aesthetically aware	4.95 ^b^	1.586		

Note: Within each variable, cluster means sharing at least one superscript letter are not significantly different from one another, whereas means with no superscript letter in common differ significantly according to Tukey’s HSD post hoc test (*p* < 0.05).

**Table 5:** Cluster preferences with respect to comfort indicators of woollen clothes.

Environmental Topic	Cluster	*N*	Mean	SD	F	*p*-Value
It must not sting	Sustainability-sensitive	88	5.02 ^b^	1.762	55.246	<0.001
	Disinterested	57	2.96 ^a^	1.991		
	Eco-aesthetic	144	5.51 ^bc^	0.996		
	Aesthetically aware	111	5.57 ^c^	0.959		
It must be warm	Sustainability-sensitive	88	5.15 ^b^	1.505	65.738	<0.001
	Disinterested	57	2.93 ^a^	1.888		
	Eco-aesthetic	144	5.51 ^b^	0.853		
	Aesthetically aware	111	5.45 ^b^	1.007		
It must be soft	Sustainability-sensitive	88	4.99 ^b^	1.615	60.962	<0.001
	Disinterested	57	2.84 ^a^	1.850		
	Eco-aesthetic	144	5.37 ^b^	0.914		
	Aesthetically aware	111	5.41 ^b^	1.021		
Not bulky	Sustainability-sensitive	88	4.25 ^b^	1.859	35.180	<0.001
	Disinterested	57	2.19 ^a^	1.807		
	Eco-aesthetic	144	4.57 ^b^	1.326		
	Aesthetically aware	111	4.63 ^b^	1.589		
Hypoallergenic	Sustainability-sensitive	88	4.49 ^b^	1.888	32.270	<0.001
	Disinterested	57	2.30 ^a^	1.908		
	Eco-aesthetic	144	4.67 ^b^	1.354		
	Aesthetically aware	111	4.68 ^b^	1.690		
	Total	400	4.29	1.844		
Elastic	Sustainability-sensitive	88	3.63 ^b^	1.834	27.126	<0.001
	Disinterested	57	1.88 ^a^	1.615		
	Eco-aesthetic	144	3.93 ^b^	1.456		
	Aesthetically aware	111	4.12 ^b^	1.656		
Breathable	Sustainability-sensitive	88	4.64 ^b^	1.648	38.265	<0.001
	Disinterested	57	2.40 ^a^	1.689		
	Eco-aesthetic	144	4.78 ^b^	1.371		
	Aesthetically aware	111	4.75 ^b^	1.516		
Washable at high temperatures	Sustainability-sensitive	88	2.52 ^b^	2.084	18.075	<0.001
	Disinterested	57	1.53 ^a^	1.627		
	Eco-aesthetic	144	2.96 ^bc^	1.621		
	Aesthetically aware	111	3.60 ^c^	1.830		
Sweat-absorbing capacity	Sustainability-sensitive	88	3.88 ^b^	1.952	22.841	<0.001
	Disinterested	57	1.89 ^a^	1.687		
	Eco-aesthetic	144	3.86 ^b^	1.741		
	Aesthetically aware	111	4.16 ^b^	1.708		
Thermoregulator	Sustainability-sensitive	88	4.50 ^b^	1.845	27.271	<0.001
	Disinterested	57	2.46 ^a^	1.763		
	Eco-aesthetic	144	4.51 ^b^	1.542		
	Aesthetically aware	111	4.68 ^b^	1.519		

Note: Within each variable, cluster means sharing at least one superscript letter are not significantly different from one another, whereas means with no superscript letter in common differ significantly according to Tukey’s HSD post hoc test (*p* < 0.05). A mean labelled with two letters (e.g., ^bc^) does not differ significantly from means sharing either letter.

**Table 6:** Knowledge of the term Ecosystem Services by cluster.

Knowledge of the Term “Ecosystem Services”	Sustainability-Sensitive	Disinterested	Eco-Aesthetic	Aesthetically Aware
Yes, I know the term	21.6%	28.1%	13.9%	13.5%
Yes, approximately	38.6%	28.1%	39.6%	27.0%
Heard it but do not know the meaning	13.6%	14.0%	27.1%	19.8%
I have never heard it	26.1%	29.8%	19.4%	39.6%

χ^2^ = 26.209, *p* = 0.002.

## Data Availability

The data that support the findings of this study are available on request from the corresponding author (V.M.M.).

## Supplementary Materials

The following supporting information can be downloaded at https://www.mdpi.com/article/10.3390/ani16172649/s1, Table S1: questionnaire framework for Section 1, Section 2 and Section 3; Table S2: framework of the questionnaire section dedicated to ES knowledge and perception (Section 4); Table S3: distribution of socio-demographic characteristics in the total sample; Table S4: multi-factor ANOVA results for ecosystem services perception by cluster.

## Abbreviations

The following abbreviations are used in this manuscript:

ESs	Ecosystem Services
PCA	Principal Component Analysis
KMO	Kaiser–Meyer–Olkin
AIC	Akaike Information Criterion
BIC	Bayesian Information Criterion
