# Supplementary material for: Linking Ecosystem-Service Perceptions and Wool Valorisation in Alpine Sheep Farming Systems
Source: Animals (Basel). 2026 Aug 24;16(17):2649. doi: 10.3390/ani16172649 (PMC13564273; doi:10.3390/ani16172649)
Supplement: Supplementary file 1 [file animals-16-02649-s001.zip › animals-4479778-supplementary.pdf]

**Table S1. Questionnaire framework for Sections 1, 2 and 3.**

| Question | Options | Measure |
|---|---|---|
| **Section 1 – Socio-demographic characteristics** | | |
| With which gender do you identify? | Men / Women / Non-binary | Closed-ended answer |
| What is your age? | 18–25 / 26–35 / 36–45 / 46–55 / >55 | Closed-ended answer |
| What is your level of education? | Primary school / Lower secondary / Upper secondary / Master's degree | Closed-ended answer |
| What is your annual family income? | < €15,000 / €16,000–25,000 / €26,000–40,000 / €41,000–60,000 / > €60,000 / Prefer not to answer | Closed-ended answer |
| Are you s resident of Lanzo Valleys? | Resident > 10 years / Resident < 10 years / Not resident | Closed-ended answer |
| What is the size of your city of residence? | Rural area / 5–10k / 10–30k / 30–60k / 60–100k / 100–250k / >250k inhabitants | Closed-ended answer |
| **Section 2 – Consumer concerns regarding certain environmental issues** | | |
| How concerned are you, in your daily life, about each of the following environmental issues? | Daily use of chemicals; Environmental impact of transport; Deforestation; Food handling; Waste increase; Depletion of natural resources; Soil, air and water pollution; Climate change; Consumeristic habits | 7-point Likert scale from 0 = not at all concerned to 6 = extremely concerned |
| **Section 3 – Wool product purchasing preferences and habits** | | |
| How often do you purchase wool products? | Often / Sometimes / Rarely / Never | Closed-ended answer |
| Which type of wool product have you purchased? | Clothing / Mattresses / Blankets / Carpets / Pillows / Underwear / Accessories | Multiple-choice answer |

| | | |
|---|---|---|
| When choosing to purchase a wool product, how important are the following comfort indicators to you? | Must not sting; Must be warm; Must be soft; Not bulky; Hypoallergenic; Elastic; Breathable; Washable at high temperatures; Sweat-absorbing; Thermoregulator | 7-point Likert scale (0 = not important – 6 = very important) |
| When choosing to purchase a wool product, how important are the following attributes to you? | Price; Quality; Aesthetics; Comfort; Made in Italy; Originality; Brand; Health safety; Environmental sustainability; Respect for working conditions; Animal welfare | 7-point Likert scale (0 = not important – 6 = very important) |
| Do you know the wool production process? | Yes / Yes, approximately / No | Closed-ended answer |

**Table S2. Framework of the questionnaire section dedicated to ES knowledge and perception (Section 4).**

| Question | Options | Measure |
|---|---|---|
| Are you familiar with the term 'Ecosystem Services'? | Yes, I know the term / Yes, approximately / I have heard the term but do not know its meaning / I have never heard it | Closed-ended answer |
| In your opinion, how important is Alpine sheep farming for providing each of the following provisioning ES? | Food source (meat, milk, cheese); Raw materials (wool, leather); Fertilisers (manure); Preservation of native/typical breeds | 7-point Likert scale (0 = not important at all – 6 = extremely important) |
| In your opinion, how important is Alpine sheep farming for providing each of the following regulating ES? | Erosion control; Water quality regeneration; Fire prevention; Control of invasive/pest species | 7-point Likert scale |
| In your opinion, how important is Alpine sheep farming for providing each of the following habitat and biodiversity ES? | Seed dispersal and plant biodiversity; Habitat preservation for other species | 7-point Likert scale |
| In your opinion, how important is Alpine sheep farming for providing each of the following cultural ES? | Preservation of typical landscape; Attraction for tourism and environmental education; Preservation of traditions and cultural heritage; Preservation of high-quality typical products | 7-point Likert scale |

| | | |
|---|---|---|
| In your opinion, how important is Alpine sheep farming for providing each of the following animal welfare aspects? | Preservation of animal welfare management practices; Protection of food chain health linked to animal welfare | 7-point Likert scale |

**Table S3. Distribution of socio-demographic characteristics in the total sample.**

| **Socio-demographic variables** | **Option** | **% total sample (n = 400)** |
|---|---|---|
| Gender | Women | 78.8 |
| | Men | 20.8 |
| | Prefer not to answer | 0.5 |
| Education level | | |
| | Primary/Lower secondary school | 9.5 |
| | Higher secondary school | 54.5 |
| | Master's degree | 36.1 |
| Age | 18–25 | 7.5 |
| | 26–35 | 13.3 |
| | 36–45 | 16.8 |
| | 46–55 | 28.3 |
| | 56–65 | 24.0 |
| | > 65 | 10.3 |
| Residence in the Lanzo Valleys | Resident > 10 years | 41.8 |
| | Not resident | 58.3 |
| City residence size | Rural area | 50.0 |
| | Cities with 5–10,000 inhabitants | 16.0 |
| | Cities with 10–30,000 inhabitants | 13.5 |
| | Cities with 30–60,000 inhabitants | 4.8 |

| | | |
|---|---|---|
| | Cities with 60–100,000 inhabitants | 1.3 |
| | Cities with 100–250,000 inhabitants | 1.0 |
| | Cities with > 250,000 inhabitants | 13.5 |
| Annual income range | < €25,000 | 30.0 |
| | €25,000–40,000 | 38.0 |
| | €40,000–60,000 | 9.0 |
| | > €60,000 | 1.0 |
| | Prefer not to answer | 22.0 |

## Table S4. Multi-factor ANOVA results for ecosystem services perception by cluster.

| Ecosystem service | Cluster | N | Average | Standard deviation | F | p-value |
|---|---|---|---|---|---|---|
| **Food source (meat, milk, cheese)** | Sustainability-sensitive | 88 | 5.38[b] | 1.187 | 88.378 | <0.001 |
| | Disinterested | 57 | 2.37[a] | 1.508 | | |
| | Eco-aesthetic | 144 | 5.24[b] | 1.140 | | |
| | Aesthetically aware | 111 | 5.17[b] | 1.257 | | |
| | **Total** | 400 | 4.84 | 1.597 | | |
| **Production of raw materials such as wool and leather** | Sustainability-sensitive | 88 | 5.26[b] | 1.208 | 91.702 | <0.001 |
| | Disinterested | 57 | 2.26[a] | 1.383 | | |
| | Eco-aesthetic | 144 | 5.12[b] | 1.041 | | |
| | Aesthetically aware | 111 | 4.86[b] | 1.271 | | |
| | **Total** | 400 | 4.67 | 1.551 | | |
| **Production of fertilisers (manure) that improve soil structure and fertility** | Sustainability-sensitive | 88 | 5.18[c] | 1.150 | 85.432 | <0.001 |
| | Disinterested | 57 | 2.19[a] | 1.381 | | |
| | Eco-aesthetic | 144 | 4.92[bc] | 1.129 | | |
| | Aesthetically aware | 111 | 4.71[b] | 1.261 | | |
| | **Total** | 400 | 4.53 | 1.546 | | |
| **Preservation of native/typical breeds, important for the genetic variability of livestock systems and for the preservation of the historical and identity heritage of a place and its typical productions** | Sustainability-sensitive | 88 | 5.66[c] | 0.693 | 176.125 | <0.001 |
| | Disinterested | 57 | 2.23[a] | 1.254 | | |
| | Eco-aesthetic | 144 | 5.31[bc] | 0.887 | | |
| | Aesthetically aware | 111 | 5.14[b] | 1.057 | | |
| | **Total** | 400 | 4.90 | 1.462 | | |
| **Erosion control through pasture turf that protects the soil from the force of rain and allows rainwater penetration** | Sustainability-sensitive | 88 | 5.58[c] | 0.673 | 199.074 | <0.001 |
| | Disinterested | 57 | 1.88[a] | 1.211 | | |
| | Eco-aesthetic | 144 | 5.33[bc] | 0.907 | | |
| | Aesthetically aware | 111 | 5.06[b] | 1.170 | | |
| | **Total** | 400 | 4.82 | 1.565 | | |
| **Regeneration and improvement of water quality through the presence of permanent grasslands** | Sustainability-sensitive | 88 | 5.44[c] | 0.842 | 161.888 | <0.001 |
| | Disinterested | 57 | 1.88[a] | 1.119 | | |
| | Eco-aesthetic | 144 | 5.20[bc] | 0.950 | | |
| | Aesthetically aware | 111 | 4.88[b] | 1.284 | | |
| | **Total** | 400 | 4.69 | 1.571 | | |
| | Sustainability-sensitive | 88 | 5.53[c] | 0.787 | 148.804 | <0.001 |
| | Disinterested | 57 | 1.86[a] | 1.060 | | |

| | | | | | | |
|---|---|---|---|---|---|---|
| **Fire prevention through the reduction of biomass and forest continuity** | Eco-aesthetic | 144 | $5.22^{bc}$ | 1.093 | | |
| | Aesthetically aware | 111 | $4.94^{b}$ | 1.397 | | |
| | **Total** | 400 | 4.73 | 1.636 | | |
| **Control of invasive and pest species** | Sustainability-sensitive | 88 | $5.45^{c}$ | 0.815 | 185.143 | <0.001 |
| | Disinterested | 57 | $1.79^{a}$ | 1.130 | | |
| | Eco-aesthetic | 144 | $5.28^{bc}$ | 0.927 | | |
| | Aesthetically aware | 111 | $4.98^{b}$ | 1.236 | | |
| | **Total** | 400 | 4.74 | 1.591 | | |
| **Dispersal of seeds in the environment and preservation of plant biodiversity** | Sustainability-sensitive | 88 | $5.55^{c}$ | 0.772 | 192.409 | <0.001 |
| | Disinterested | 57 | $1.96^{a}$ | 1.210 | | |
| | Eco-aesthetic | 144 | $5.31^{bc}$ | 0.822 | | |
| | Aesthetically aware | 111 | $5.05^{b}$ | 1.159 | | |
| | **Total** | 400 | 4.81 | 1.526 | | |
| **Preservation of habitats for other animal species such as migratory birds, insects, reptiles and large mammals also through path cleaning** | Sustainability-sensitive | 88 | $5.49^{c}$ | 0.816 | 231.441 | <0.001 |
| | Disinterested | 57 | $1.82^{a}$ | 1.054 | | |
| | Eco-aesthetic | 144 | $5.38^{bc}$ | 0.756 | | |
| | Aesthetically aware | 111 | $5.11^{b}$ | 1.139 | | |
| | **Total** | 400 | 4.82 | 1.542 | | |
| **Preservation of a typical landscape (pastures and hamlets)** | Sustainability-sensitive | 88 | $5.52^{b}$ | 0.802 | 182.376 | <0.001 |
| | Disinterested | 57 | $1.96^{a}$ | 1.133 | | |
| | Eco-aesthetic | 144 | $5.35^{b}$ | 0.872 | | |
| | Aesthetically aware | 111 | $5.17^{b}$ | 1.242 | | |
| | **Total** | 400 | 4.86 | 1.557 | | |
| **Attraction for wine and food, sports, ecological tourism and environmental education** | Sustainability-sensitive | 88 | $5.01^{b}$ | 1.150 | 130.294 | <0.001 |
| | Disinterested | 57 | $1.67^{a}$ | 0.893 | | |
| | Eco-aesthetic | 144 | $4.97^{b}$ | 1.158 | | |
| | Aesthetically aware | 111 | $4.90^{b}$ | 1.300 | | |
| | **Total** | 400 | 4.49 | 1.636 | | |
| **Preservation of the area's traditions and historical-cultural heritage** | Sustainability-sensitive | 88 | $5.50^{c}$ | 0.830 | 210.942 | <0.001 |
| | Disinterested | 57 | $1.91^{a}$ | 0.931 | | |
| | Eco-aesthetic | 144 | $5.40^{bc}$ | 0.804 | | |
| | Aesthetically aware | 111 | $5.13^{b}$ | 1.214 | | |
| | **Total** | 400 | 4.85 | 1.538 | | |
| **Preservation of high-quality typical products** | Sustainability-sensitive | 88 | $5.60^{b}$ | 0.720 | 222.006 | <0.001 |
| | Disinterested | 57 | $2.09^{a}$ | 1.023 | | |
| | Eco-aesthetic | 144 | $5.44^{b}$ | 0.764 | | |
| | Aesthetically aware | 111 | $5.28^{b}$ | 1.130 | | |
| | **Total** | 400 | 4.95 | 1.483 | | |
| **Preservation of management practices that respect animal welfare** | Sustainability-sensitive | 88 | $5.47^{c}$ | 0.909 | 191.282 | <0.001 |
| | Disinterested | 57 | $1.86^{a}$ | 0.833 | | |
| | Eco-aesthetic | 144 | $5.36^{c}$ | 0.706 | | |
| | Aesthetically aware | 111 | $4.95^{b}$ | 1.407 | | |
| | **Total** | 400 | 4.77 | 1.566 | | |
| **Protection of the health and safety of the food chain directly linked to animal welfare** | Sustainability-sensitive | 88 | $5.61^{c}$ | 0.794 | 212.074 | <0.001 |
| | Disinterested | 57 | $1.98^{a}$ | 1.009 | | |
| | Eco-aesthetic | 144 | $5.38^{bc}$ | 0.748 | | |
| | Aesthetically aware | 111 | $5.09^{b}$ | 1.210 | | |
| | **Total** | 400 | 4.87 | 1.518 | | |

Note: Different superscript letters ($^{a}$, $^{b}$, $^{c}$) within the same row indicate statistically significant differences between cluster means according to Tukey's HSD post hoc test ($p < 0.05$).
